# Effects of *Moringa oleifera* Lam. Supplementation on Cardiometabolic Outcomes: A Meta-Analysis of Randomized Controlled Trials with GRADE Assessment

**DOI:** 10.3390/nu17223501

**Published:** 2025-11-07

**Authors:** Diana Crișan, Laura Gavrilaș, Ramona Păltinean, Oleg Frumuzachi, Andrei Mocan, Gianina Crișan

**Affiliations:** 1 Department of Pharmaceutical Botany, Faculty of Pharmacy, “Iuliu Hațieganu” University of Medicine and Pharmacy, 23 Gheorghe Marinescu Street, 400337 Cluj-Napoca, Romania; diana.suciu.crisan@elearn.umfcluj.ro (D.C.); rpaltinean@umfcluj.ro (R.P.); mocan.andrei@umfcluj.ro (A.M.); gcrisan@umfcluj.ro (G.C.); 2 Department 2, Faculty of Nursing and Health Sciences, “Iuliu Hațieganu” University of Medicine and Pharmacy, 23 Gheorghe Marinescu Street, 400337 Cluj-Napoca, Romania; laura.gavrilas@umfcluj.ro

**Keywords:** phytotherapy, functional foods, herbal medicine, blood glucose regulation, supplementation

## Abstract

**Background/Objectives:** *Moringa oleifera* Lam. is traditionally used in African and Asian medicine for its nutritional and therapeutic properties. Rich in (poly)phenols, vitamins, and minerals, various parts of the plant were used to manage metabolic disorders. No systematic research critically evaluated its impact on cardiometabolic outcomes assessed through randomized controlled trials (RCTs), thus this study aimed to do so by synthesizing existing evidence. **Methods:** A systematic search of PubMed, Scopus, and Web of Science databases was conducted through 7 April 2025. Eligible RCTs with a minimum duration of two weeks that evaluated moringa supplementation in adults (≥18 years) and reported at least one cardiometabolic outcome, including anthropometric measures, lipid profile, glycemic indices, or blood pressure. Random-effects meta-analyses were performed using standardized mean differences, risk of bias was assessed using the Cochrane RoB 2 tool, while the certainty of evidence was evaluated using GRADE assessment. **Results:** Nine RCTs (12 study arms) involving 341 participants in the intervention and 308 in the control groups met inclusion criteria. Moringa supplementation showed no significant effects on most considered outcomes. A modest reduction in diastolic blood pressure (SMD: −0.41; 95% CI: −0.75 to −0.07; *p* = 0.02; *I*^2^ = 19%) was observed; however, this effect was not robust in sensitivity analyses. Subgroup analyses suggested potential benefits at doses <10 g/day, in participants <50 years old, and in interventions lasting ≥12 weeks. Nevertheless, risk of bias, substantial heterogeneity (*I*^2^ frequently >80% for anthropometric and lipid parameters), indirectness, and methodological limitations reduced the overall certainty of evidence to very low for all the evaluated outcomes. **Conclusions:** Current evidence does not support consistent cardiometabolic benefits of moringa supplementation in adults. Further large-scale, rigorously designed RCTs are warranted to clarify its therapeutic potential and optimal supplementation parameters.

## 1. Introduction

Non-communicable diseases (NCDs) are the leading cause of death globally, accounting for approximately 75% of non-pandemic-related deaths globally, with cardiometabolic disorders, such as cardiovascular disease, type 2 diabetes, dyslipidemia, and hypertension, representing the most significant contributors [1]. A substantial portion of this global burden is attributable to modifiable metabolic risk factors, including increased fasting blood glucose (FBG), total cholesterol (TC), low-density lipoprotein cholesterol (LDL-C), and triglycerides (TAGs) [2,3,4]. These metabolic risk factors are frequently accompanied by anthropometric changes (i.e., increased body weight [BW], body mass index [BMI], waist circumference [WC], and excess adiposity) and elevated blood pressure (systolic and diastolic) [5,6,7]. According to current clinical guidelines from organizations such as the American Heart Association and the International Diabetes Federation, these parameters are recognized as critical indicators of cardiometabolic risk, as they contribute to insulin resistance, endothelial dysfunction, and atherogenesis, key processes underlying the development of cardiometabolic diseases [8,9].

Given the growing burden of these conditions, the development of preventive and supportive therapeutic strategies is critical. Among these, nutritional interventions, including the use of functional foods and nutraceuticals, gained increasing attention for their role in modulating key cardiometabolic risk factors, such as fasting blood glucose, lipid profile, blood pressure, and anthropometric indices [10,11,12,13,14].

*Moringa oleifera* Lam. (commonly referred to as moringa, hereafter used as such unless otherwise specified) is a plant native to the Indian subcontinent, particularly Afghanistan, Bangladesh, India, and Pakistan. Nowadays, it is globally recognized for its high nutritional value and medicinal properties [15]. Moringa leaves, seeds, pods, and bark are rich in bioactive compounds, including flavonoids, glucosinolates, alkaloids, carotenoids, and essential amino acids, along with a high content of vitamins (A, C, E, and B-complex), minerals (calcium, potassium, iron), and fiber [15]. These phytochemicals were associated with antioxidant [16], anti-inflammatory [17,18], antidiabetic [19,20], and lipid-lowering activities [21,22], positioning moringa as a valuable ingredient for functional food development and the nutritional management of cardiometabolic diseases [23].

In accordance, several meta-analyses in animal models reported beneficial effects of moringa supplementation, including improvements in lipid profiles and glycemic control [24,25,26]. However, despite the encouraging findings from preclinical studies, RCTs in humans yielded mixed results, with some reporting benefits on glycemic control and lipid levels [27,28,29], while others observed no significant effects [30,31]. A meta-analysis of five small randomized and non-randomized trials involving patients with type 2 diabetes mellitus or prediabetes found no significant effects of moringa supplementation on HbA1c or fasting glucose [32]. Nonetheless, all outcomes were rated as low to very-low certainty due to methodological limitations and study heterogeneity.

Given the methodological limitations and existing knowledge gaps of previous meta-analyses, which included non-randomized trials, this meta-analysis aimed to systematically evaluate and quantify the effects of moringa supplementation, based exclusively on randomized controlled trials (RCTs), on cardiometabolic outcomes in adults (≥18 years), irrespective of baseline health status, including populations with metabolic disorders or other chronic conditions such as HIV infection. Additionally, we conducted the risk of bias assessment and graded the certainty of evidence to evaluate the overall quality and strength of the available evidence. This approach aimed to increase internal validity and provide a more comprehensive synthesis of evidence across diverse adult populations.

## 2. Materials and Methods

### 2.1. Protocol and Registration

This systematic review and meta-analysis were conducted in accordance with the Preferred Reporting Items for Systematic Reviews and Meta-Analyses (PRISMA) 2020 guidelines [33] and was prospectively registered in PROSPERO (registration number: CRD420251025219).

### 2.2. Literature Search Strategy and Eligibility Criteria

Three databases (PubMed, Scopus, Web of Science) were searched from inception to 7 April 2025. The search strategy included terms related to *M. oleifera* and cardiometabolic health outcomes (e.g., “Moringa oleifera”, “lipid profile”, “blood pressure”, “glucose”, “insulin”, “HbA1c”, “cholesterol”). No restrictions were applied to language or publication status. Additional studies were identified by screening the reference lists of included articles and reviewing articles that cited relevant studies (“snowballing”). Authors were contacted to obtain additional data when necessary. The detailed search strings for each database are provided in Supplementary Table S1.

The research question and eligibility criteria were defined using the PICOS framework (Table 1), specifying population, intervention, comparator, outcomes, and study design characteristics relevant to cardiometabolic effects of moringa. Studies involving multi-component interventions where the effect of moringa could not be isolated, conducted in children (<18 years old) or pregnant/lactating women (physiological changes during these periods can alter cardiometabolic responses and nutrient metabolism, potentially confounding the effects of supplementation), non-randomized or preclinical studies, and those lacking a control group or reporting insufficient outcome data were excluded. Also, reviews, study protocols, conference abstracts, and case reports were excluded, as were trials with a duration of <2 weeks. Furthermore, studies were excluded if they did not report mean changes with corresponding standard deviations (SDs), or did not provide enough information to compute them, such as baseline and final means with SDs, confidence intervals, standard errors, *p*-values, or sample sizes for each group.

### 2.3. Study Selection and Data Extraction

Two reviewers independently screened titles and abstracts, followed by full-text articles, using the predefined inclusion criteria. Discrepancies were resolved through discussion; if consensus could not be reached, a third reviewer was consulted to achieve agreement. A structured data extraction form was used to systematically collect information from each study. Extracted data included author(s) and year of publication, country where the study was conducted, study design (parallel or crossover; blinding), characteristics of participants (including health status, age, sex distribution, and baseline BMI), details of the intervention and control groups (form of moringa used, dosage, nutrient or (poly)phenol content when reported), total sample size and group allocation, intervention duration, and all reported cardiometabolic outcomes. For each included study, data on pre- and post-intervention values for the selected outcomes were extracted, including either mean changes with standard deviations (SDs) or sufficient information to calculate them. In trials with multiple intervention groups (e.g., differing moringa dosages) that shared a single control group, the control group was used as a common comparator for all intervention arms. Separate effect sizes were calculated for each intervention arm compared to the shared control, in accordance with the study design.

### 2.4. Risk of Bias Assessment and Certainty of Evidence

The risk of bias for each included study was assessed using Version 2 of the Cochrane Risk of Bias tool for randomized trials (RoB 2), which is the recommended instrument for evaluating randomized controlled trials in Cochrane Reviews [34]. RoB 2 is organized into five domains, each focusing on a specific aspect of trial design, conduct, or reporting: (1) bias arising from the randomization process, (2) bias due to deviations from intended interventions, (3) bias due to missing outcome data, (4) bias in measurement of the outcome, and (5) bias in selection of the reported result. Within each domain, a series of signaling questions guided the evaluators in collecting relevant information about the study features. Based on responses to these signaling questions, an algorithm generated a proposed judgment for each domain: ‘Low risk of bias’, ‘Some concerns’, or ‘High risk of bias’. An overall risk of bias judgment was then assigned to each study, following the Cochrane guidance.

The certainty of the evidence was evaluated for each primary outcome separately. The GRADE (Grading of Recommendations Assessment, Development and Evaluation) approach [35] was applied. Downgrading was based on predefined domains (1) risk of bias, (2) inconsistency, (3) indirectness, (4) imprecision, and (5) other considerations. We started RCT evidence at high certainty and downgraded by one or two levels per GRADE guidance. The final certainty of evidence for each outcome was rated as high, moderate, low, or very low.

Two evaluators independently assessed risk of bias assessment and certainty of evidence, and any discrepancies in judgment were resolved through discussion or consultation with a third evaluator.

### 2.5. Statistical Analysis

Mean difference in change and corresponding standard deviations (SDs) for each outcome were extracted from both intervention and control groups. In cases where these values were not directly reported, they were estimated from baseline (*M_b_*, *SD_b_*) and post-intervention (*M_f_*, *SD_f_*) values using the following equations: $Mean=\;M_{b}-M_{f}$ and $SD=\surd ({SD}_{b}^{2}+{SD}_{f}^{2})-(2\times R\times {SD}_{b}\times {SD}_{f}$), with a default correlation coefficient (*R*) of 0.5. To assess the robustness of these imputations, sensitivity analyses were conducted using alternative *R* values of 0.2 and 0.8 [36]. When studies reported the standard error of the mean (*SEM*) rather than *SD*, the *SD* was calculated as SD=SEM×√n, where *n* represents the group sample size.

Meta-analyses were performed using the standardized mean difference (SMD) with 95% confidence intervals (CIs) as the measure of effect size. Between-study heterogeneity was evaluated using the *I*^2^ statistic, with thresholds of 25%, 50%, and 75% interpreted as low, moderate, and high heterogeneity, respectively. Owing to the limited number of studies (<10 per outcome), assessment of publication bias (e.g., via funnel plots or Egger’s test) and meta-regression analyses were not feasible, as such models would lack sufficient statistical power and could yield unreliable estimates. Instead, prespecified subgroup analyses (by moringa dose [<10 g/day vs. ≥10 g/day], intervention duration [<12 weeks vs. ≥12 weeks], participant age [<50 years vs. ≥50 years], and baseline BMI [<25 kg/m^2^ vs. ≥25 kg/m^2^]) were used to explore potential sources of heterogeneity. To account for potential confounding effects, sensitivity analyses were conducted for each outcome, including and excluding trials, to evaluate their influence on the pooled estimates. All statistical analyses were conducted using Review Manager (RevMan) version 5.4 and RStudio version 2024.04.2 + 764 (RStudio PBC, Boston, MA, USA).

## 3. Results

### 3.1. Literature Search and Studies’ Characteristics

The systematic search of electronic databases identified a total of 735 records: PubMed (*n* = 62), Web of Science (*n* = 454), and Scopus (*n* = 219). After removing 127 duplicates, 608 unique records remained for title and abstract screening. Of these, 587 were excluded as they were review articles, animal studies, or studies involving pregnant or lactating women, children, acute interventions, in vitro experiments, or cell-based assays.

**Figure 1:**
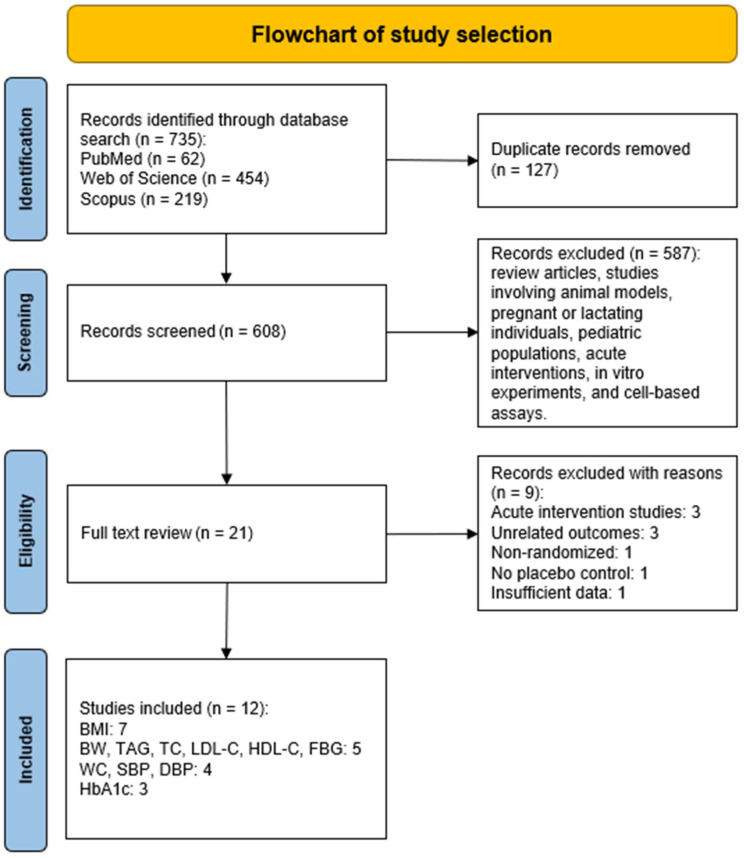
PRISMA 2020 flow diagram illustrating the literature search, screening, eligibility assessment, and inclusion process for randomized controlled trials evaluating the effects of *Moringa oleifera* supplementation on cardiometabolic outcomes in adults.

Twenty-one full-text articles were assessed for eligibility. Nine of them were excluded for the following reasons: acute intervention design (*n* = 3) [37,38,39], unrelated outcomes (*n* = 3) [40,41,42], lack of randomization (*n* = 1) [43], absence of a proper placebo control group (*n* = 1) [44], or insufficient data reporting (*n* = 1) [45]. Ultimately, 12 publications corresponding to 9 unique RCTs met the eligibility criteria and were included in the meta-analysis. These trials contributed data to the following outcomes: BMI (*n* = 7); BW, TAGs, TC, LDL-C, HDL-C, and FBG (*n* = 5 each); WC, SBP and DBP (*n* = 4 each); and HbA1c (*n* = 3). The study selection process is illustrated in the PRISMA flow diagram (Figure 1).

Notably, one trial was published in three separate publications [46,47,48], all describing the same study protocol, population, and outcomes; these were treated as a single study. Another RCT was reported in two complementary publications [27,28], which presented non-overlapping outcomes derived from the same cohort; these were combined and treated as a single study arm for analysis.

The characteristics of all included RCTs are summarized in Table 2. All included studies evaluating the effects of moringa supplementation on cardiometabolic outcomes employed a parallel-group design; none were crossover trials. Among these trials, four were double-blinded [27,28,29,30,46,47,48], one was single-blinded [49], one was unblinded [50], and the blinding status was unclear in three studies [31,51,52].

The studies were published between 2017 [31,49] and 2025 [50,52] and conducted across seven countries: Democratic Republic of the Congo [49], India [29], Italy [50], Nigeria [30,46,47,48], Pakistan [51,52], Spain [27,28], and Thailand [31]. The included populations (intervention *n* = 341, control *n* = 308) were heterogeneous and included adults with prediabetes or type 2 diabetes mellitus (T2DM) [27,28,30,31,50], HIV-infected individuals [46,47,48,49], overweight individuals [29,52], and patients with hyperlipidemia [51]. The mean age of participants ranged from ~36.3 [46,47,48] to 61 years [50], while the mean baseline BMI ranged from 21.4 [49] to 32.50 kg/m^2^ [51], indicating a population predominantly affected by overweight or obesity.

Moringa was administered in various formulations, most commonly as leaves powder [27,28,31,46,47,48,49,50,51,52]. One study used steamed moringa leaves [30], and another employed a standardized polyherbal formulation containing hydro-ethanolic moringa leaves extract combined with *Murraya koenigii* (L.) Spreng. and *Curcuma longa* L. [29]. The daily dosage ranged from 900 mg/d of extract [29] or 1 g/day of leaves powder [52] to 60 g/day of leaves powder [30], depending on the formulation and study protocol. The duration of interventions ranged from 2 weeks [30] to 6 months [46,47,48,49].

### 3.2. Moringa Supplementation Impact on Anthropometric Parameters

**Figure 2:**
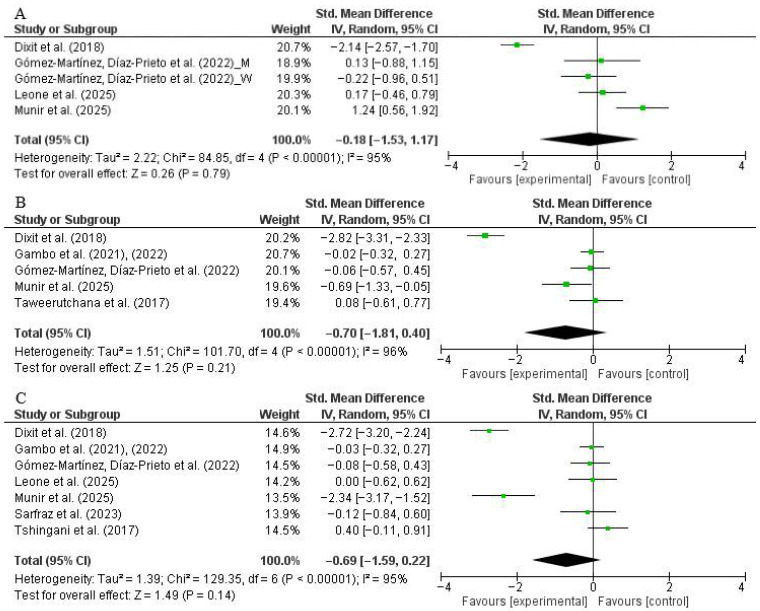
Forest plot representation of randomized controlled trials evaluating the impact of *Moringa oleifera* supplementation on anthropometric parameters ((**A**): body weight, (**B**): body mass index, (**C**): waist circumference). No significant effects were found for any anthropometric outcome (*p* > 0.05), with high heterogeneity across analyses (*I*^2^ = >90%). Sensitivity analyses confirmed that no single study disproportionately influenced the pooled results. Also, subgroup analyses by dose, age, and BMI did not show significant impact of moringa supplementation on anthropometric parameters [27,28,29,31,46,47,48,49,50,51,52].

A total of five trials (intervention: *n* = 220; control: *n* = 219) reported on changes in BW following moringa supplementation. The meta-analysis showed a non-significant reduction in BW compared to placebo, with a pooled SMD of −0.70 (95% CI: −1.81 to 0.40, *p* = 0.21; *I*^2^ = 96%) (Figure 2A). Sensitivity analyses excluding one study at a time yielded consistent findings, suggesting that no single study had a disproportionate influence on the pooled result. Subgroup analysis by age did not indicate significant reduction in BW among <50 years participants compared to those ≥50 years (Supplementary Table S2).

A total of seven trials (intervention: *n* = 279; control: *n* = 263) assessed BMI. The pooled analysis showed a non-significant reduction in BMI (SMD: −0.69, 95% CI: −1.59 to 0.22, *p* = 0.14; *I*^2^ = 95%) (Figure 2B). Sensitivity analysis again confirmed the robustness of the findings. Subgroup analysis by dosage, age, and BMI did not provide meaningful results (Supplementary Table S2).

Regarding WC, four trials (intervention: *n* = 135; control: *n* = 125) were included. The overall effect was again non-significant (SMD: −0.18, 95% CI: −1.53 to 1.17, *p* = 0.79; *I*^2^ = 95%) (Figure 2C). Sensitivity analysis confirmed that the pooled result was not affected by the exclusion of any single study. Subgroup analysis by age showed no substantial differences in outcomes between participants <50 years and those ≥50 years (Supplementary Table S2).

Overall, moringa supplementation did not significantly improve anthropometric outcomes compared to placebo, and the results were marked by considerable heterogeneity. Subgroup analyses suggested possible effect modification by age, dosage, and BMI category, though none reached statistical significance (Supplementary Table S2).

### 3.3. Moringa Supplementation Impact on Lipid Parameters

**Figure 3:**
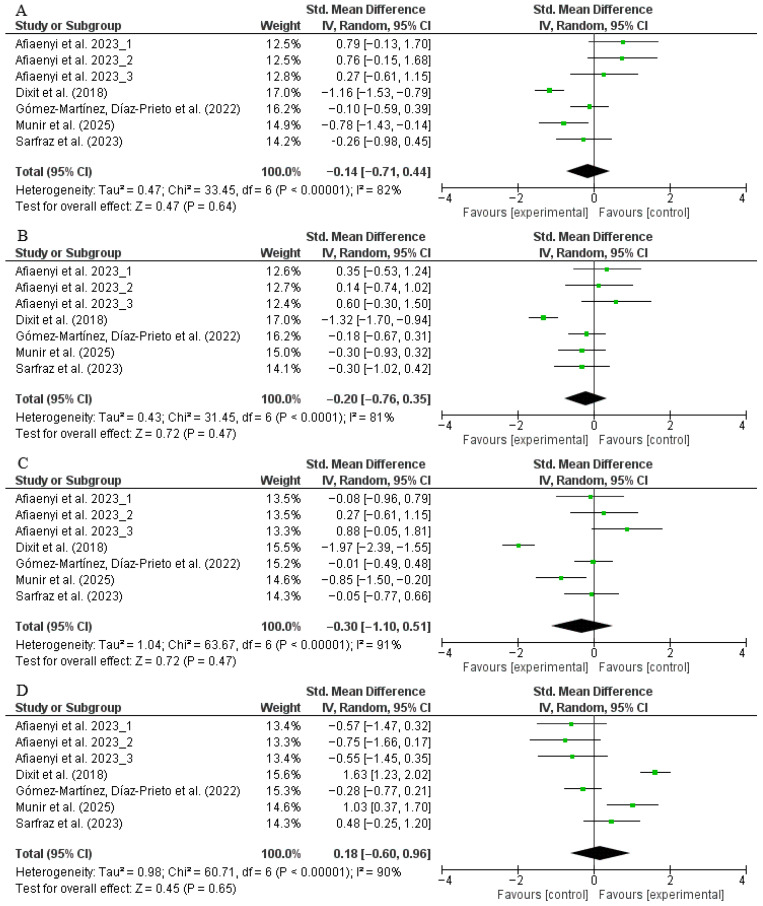
Forest plot representation of randomized controlled trials evaluating the impact of *Moringa oleifera* supplementation on lipid profile ((**A**): triacylglycerols, (**B**): total cholesterol, (**C**): LDL-C, (**D**): HDL-C). No significant pooled effects were observed for any lipid outcome (*p* > 0.05), and heterogeneity remained substantial (*I*^2^ = 81–91%). Subgroup analyses suggested potential significant improvements in TAGs by dosage, intervention duration, and participant age. Likewise, HDL-C increased in <50-year-old participants, while those ≥50 years and participants receiving ≥10 g/day experienced a significant reduction [27,28,29,30,51,52].

A total of five trials (intervention: *n* = 162; control: *n* = 163) assessed changes in TAGs levels. The meta-analysis showed no significant effect of moringa supplementation compared to placebo, with a pooled SMD of −0.14 (95% CI: −0.71 to 0.44, *p* = 0.64; *I*^2^ = 82%) (Figure 3A). Sensitivity analyses confirmed the robustness of this result. Subgroup analysis showed a significant reduction in TAGs in participants consuming <10 g/day, while no effect was observed in those receiving ≥10 g/day, with a statistically significant difference between dosage groups (*p* = 0.002). Participants receiving ≥12 weeks of supplementation also showed greater TAGs reduction compared to <12 weeks (*p* = 0.02). Subgroup analysis by age showed a trend toward greater effect in <50-year-old participants (*p* = 0.002) (Supplementary Table S2).

For TC, data were available from five trials (intervention: *n* = 162; control: *n* = 163). The pooled analysis showed no significant effect of supplementation (SMD: −0.20, 95% CI: −0.76 to 0.35, *p* = 0.47; *I*^2^ = 81%) (Figure 3B). Sensitivity analysis confirmed the stability of the pooled estimate. Subgroup analysis by dosage showed a more favorable effect at <10 g/day compared to ≥10 g/day, with a statistically significant difference between subgroups (*p* = 0.03). No more significant differences among intervention duration, participant age, and baseline BMI were noticed (Supplementary Table S2).

Five trials (intervention: *n* = 162; control: *n* = 163) also assessed LDL-C, with a pooled SMD of −0.30 (95% CI: −1.10 to 0.51, *p* = 0.47; *I*^2^ = 91%) (Figure 3C). The intervention did not produce a statistically significant reduction in LDL-C levels compared to control. Sensitivity analysis did not change the results significantly. Subgroup analyses by dosage, intervention duration, participant age, and baseline BMI did not reach statistical significance (Supplementary Table S2).

Finally, for HDL-C, five trials (intervention: *n* = 162; control: *n* = 163) were included. No significant effect was found (SMD: 0.18, 95% CI: −0.60 to 0.96, *p* = 0.65; *I*^2^ = 90%) (Figure 3D). Sensitivity analysis also did not change the results significantly. Interestingly, age-stratified analysis revealed a significant improvement in HDL-C among participants <50 years old, while those ≥50 years experienced a significant reduction, with a statistically significant difference between groups (*p* = 0.02). Moreover, subgroup analysis by dosage showed a statistically significant reduction in HDL-C among participants receiving ≥10 g/day, while <10 g/day had a positive but non-significant effect. Regarding duration, trials ≥12 weeks showed a greater increase in HDL-C compared to those <12 weeks, but the subgroup difference was not statistically significant (*p* = 0.11) (Supplementary Table S2).

### 3.4. Moringa Supplementation Impact on Glycaemic Parameters

**Figure 4:**
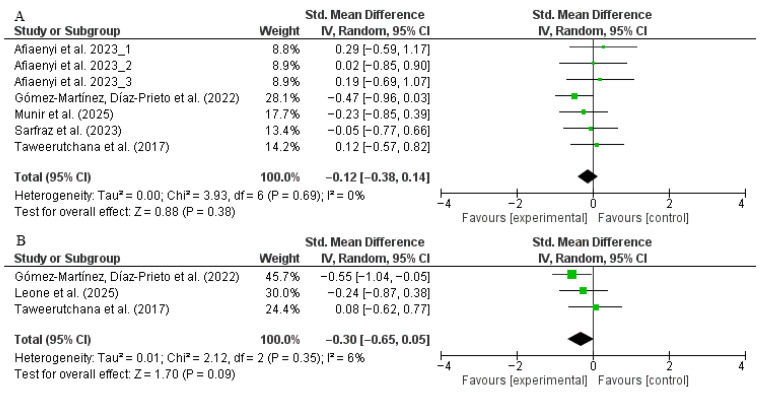
Forest plot representation of RCTs evaluating the impact of *Moringa oleifera* supplementation on glycemic parameters ((**A**): fasting blood glucose, (**B**): HbA1c). Sensitivity and subgroup analyses did not change the results significantly [27,28,30,31,50,51,52].

A total of seven trials (intervention: *n* = 112; control: *n* = 115) reported data on FBG. The pooled analysis indicated no statistically significant difference between moringa and placebo groups (SMD: −0.12, 95% CI: −0.38 to 0.14, *p* = 0.38; *I*^2^ = 0%) (Figure 4A). Sensitivity analyses revealed no change in the pooled effect when individual studies were sequentially excluded. No significant changes were noticed after conducting subgroup analyses (Supplementary Table S2).

Only three trials (intervention: *n* = 77; control: *n* = 65) evaluated the effects of moringa on HbA1c. The meta-analysis showed that moringa supplementation did not reduce HbA1c significantly (Figure 4B). No sensitivity and subgroup analyses were performed because of the limited number of trials reporting HbA1c.

### 3.5. Moringa Supplementation Impact on Blood Pressure

**Figure 5:**
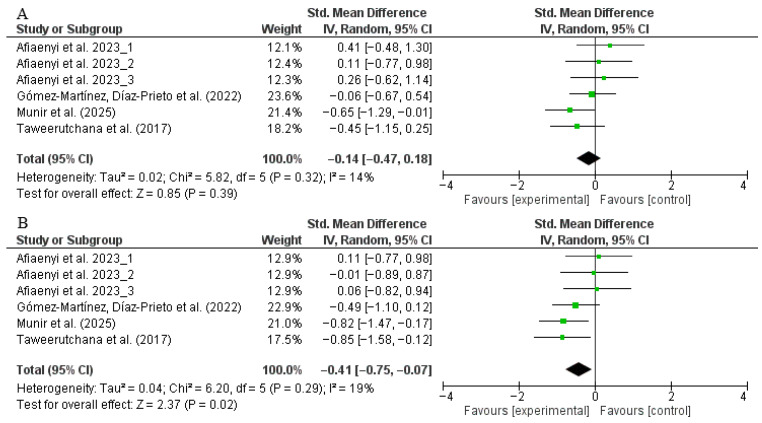
Forest plot representation of RCTs evaluating the impact of *Moringa oleifera* supplementation on blood pressure ((**A**): systolic blood pressure, (**B**): diastolic blood pressure). A modest reduction in diastolic blood pressure (DBP) was initially significant (SMD −0.41; 95% CI −0.75 to −0.07; *p* = 0.02; *I*^2^ = 19%); however, this effect was not sustained after sensitivity analysis excluding cited studies [27,28,31,52], indicating limited robustness of the finding. Subgroup analyses showed that DBP decreased significantly in trials lasting ≥12 weeks and when participants received <10 g/day of moringa. SBP did not decrease significantly in subgroup analyses [27,28,30,31,52].

Four trials (intervention: *n* = 84; control: *n* = 92) assessed the effect of moringa supplementation on SBP. The pooled analysis showed no significant difference between the intervention and placebo groups (SMD: −0.14, 95% CI: −0.47 to 0.18, *p* = 0.39; *I*^2^ = 14%) (Figure 5A). Sensitivity analysis confirmed the robustness of the result, as exclusion of individual studies did not alter the overall estimate. Subgroup analysis by dosage suggested a slight reduction in SBP with doses <10 g/day compared to ≥10 g/day, but this difference was not statistically significant (*p* = 0.05). Stratification by intervention duration (<12 vs. ≥12 weeks) showed similarly non-significant results (*p* = 0.32) (Supplementary Table S2).

A total of four trials (intervention: *n* = 84; control: *n* = 92) also reported on DBP. The meta-analysis revealed a statistically significant reduction in DBP in participants receiving moringa supplementation compared to placebo (SMD: −0.41, 95% CI: −0.75 to −0.07, *p* = 0.02; *I*^2^ = 19%) (Figure 5B). However, sensitivity analysis indicated that this effect was not robust; the statistical significance was lost when any of the following studies were excluded: [27,28,31,52]. Subgroup analysis showed that participants receiving <10 g/day had a significant reduction in DBP, while those receiving ≥10 g/day experienced no effect. Similarly, trials lasting ≥12 weeks showed a statistically significant reduction in DBP, whereas those <12 weeks did not. These findings suggest that longer interventions at lower dosages may be more effective in lowering diastolic pressure (Supplementary Table S2).

### 3.6. Risk of Bias and Certainty of the Evidence Assessment

**Figure 6:**
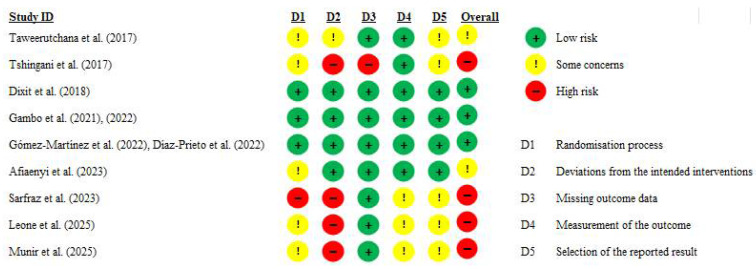
Risk of bias visualization created using RoB 2 tool [27,28,29,30,31,46,47,48,49,50,51,52].

The risk of bias in the included studies was evaluated using Version 2 of the Cochrane Risk of Bias tool for randomized trials (RoB 2), which assesses five domains: the randomization process (D1), deviations from intended interventions (D2), missing outcome data (D3), measurement of the outcome (D4), and selection of the reported result (D5). Out of the nine included trials, three studies [27,28,29,46,47,48] were judged to have overall low risk of bias across all domains. Two studies [30,31] were rated as having some concerns, primarily due to issues in the randomization process (D1), potential deviations from intended interventions (D2), or selective reporting (D5). The remaining four studies [49,50,51,52] were assessed as having a high risk of bias, largely due to concerns in multiple domains, including deviations from intended interventions (D2), issues in the randomization process (D1) and missing outcome data (D3). The summary of the risk of bias assessment is presented in Figure 6.

Most frequent issues included inadequate reporting of randomization methods, absence of blinding, and uncertainty in selective reporting when protocols were missing or retrospective. While objective laboratory measures were generally used across studies (minimizing measurement bias), the risk from domain 2 (deviations from intended interventions) and domain 5 (selection of the reported result) were most commonly increased. Detailed justifications for the risk of bias judgments across all domains for each study are presented in Supplementary Table S3.

According to the GRADE assessment (Table 3), the overall certainty of evidence for all evaluated outcomes was rated as very low. Across the included trials, all outcomes were downgraded for risk of bias due to methodological limitations, high or very high heterogeneity in effect estimates, and wide confidence intervals overlapping both potential benefit and null effect. Indirectness was generally not a concern, as interventions and populations were aligned with the review question. However, all outcomes were informed by a small number of RCTs (<10 per parameter), preventing reliable assessment of publication bias, and most studies lacked dietary monitoring or control, which may have influenced metabolic responses. Taken together, these factors indicate that the current body of evidence provides very limited confidence in the estimated effects of moringa supplementation on cardiometabolic parameters in adults.

## 4. Discussion

Moringa, often referred to as the “miracle tree,” is considered one of the most affordable and dependable sources of essential nutrients. Almost every part of the plant is utilized for its nutritional value, with the leaves being particularly rich in beta-carotene, minerals such as calcium and potassium, and macronutrients. While in vitro and in vivo animal studies have demonstrated promising effects of moringa in reducing cardiometabolic risk factors, evidence from human clinical trials remains limited and inconsistent. A prior meta-analysis evaluating the impact of moringa supplementation showed that while postprandial glucose and blood pressure were modestly improved, no significant effects were observed on key glycemic markers such as HbA1c and fasting plasma glucose [32]. However, this analysis was limited by several important methodological factors. Notably, it included both randomized and non-randomized studies, reducing the internal validity of the pooled results. Among the included studies, only three were RCTs, and one of these had methodological concerns [45], further limiting the robustness, statistical strength, and generalizability of the conclusions. Furthermore, the overall certainty of evidence was rated as low to very low due to factors such as open-label designs, heterogeneous dosing, short intervention durations (4–12 weeks), and lack of standardization in the moringa preparations used [32].

In light of these concerns, the systematic review and meta-analysis presented herein synthesized findings from 12 RCTs; nonetheless, the current body of evidence is still insufficient to draw definitive conclusions regarding moringa efficacy in adults. GRADE assessment showed that all outcomes were supported by very low certainty evidence. High or unclear risk of bias in several trials, mainly due to poor randomization reporting, absence of blinding, and selective outcome reporting, likely contributed to the inconsistency and fragility of pooled estimates. Together with substantial heterogeneity and imprecision, these factors limit the confidence in the overall findings and indicate that the observed effects of *Moringa oleifera* supplementation should be interpreted with caution.

### 4.1. Moringa Supplementation and Anthropometric Parameters

Although the pooled analysis did not demonstrate statistically significant reductions in BW, BMI, or WC, several trials reported individual improvements, especially when moringa supplementation was combined with dietary or lifestyle interventions. For instance, Munir et al. [52] observed significant reductions in all three anthropometric indices following 12 weeks of supplementation with 1 g/day of moringa leaves powder in overweight participants with hyperlipidemia [52]. However, these effects were accompanied by reduced total energy and fat intake alongside increased fiber consumption, suggesting that the observed improvements may have resulted from modifications in participants’ background diet rather than from moringa supplementation itself [52].

Conversely, Leone et al. [50], Gambo et al. [46,47,48], and Tshingani et al. [49] reported no significant changes in weight or BMI among T2DM and HIV-positive populations, despite three to six months of supplementation [46,47,48,49,50]. These null findings may reflect nutritional adequacy in the populations studied or insufficient baseline metabolic burden.

Nonetheless, preclinical studies consistently report anti-obesity and lipid-modulating effects of moringa, attributed to compounds such as chlorogenic acid, isothiocyanates, and quercetin, which may influence adipogenesis, lipase activity, and appetite-regulating hormones [26,53,54,55,56,57]. While these mechanisms support a biological rationale for potential benefits, as it was shown, they have not been translated into consistent clinical effects in humans. The substantial heterogeneity observed in the pooled analyses (*I*^2^ typically >90%) likely reflects differences in study populations, baseline BMI, intervention duration, dosage, and moringa formulations (e.g., leaf powder, steamed leaves). Such variability may have diluted potential treatment effects, contributing to the null pooled outcomes, highlighting the need for rigorously designed, adequately powered trials using standardized moringa preparations and harmonized outcome reporting to better evaluate its true impact on anthropometric parameters.

### 4.2. Moringa Supplementation and Lipid Profile

This meta-analysis did not find statistically significant effects of moringa supplementation on serum lipid parameters, including TAGs, TC, LDL-C, and HDL-C, when compared to placebo. These results might be explained by the way included studies were conducted. For instance, Dixit et al. [29] demonstrated improvements in all lipid parameters (TAGs, TC, LDL-C, and HDL-C) following a 16-week supplementation with a polyherbal formulation containing 900 mg/day of moringa alongside *M. koenigii* and *C. longa*, combined with dietary and physical activity advice. While these results appear promising, the multi-component design makes it impossible to attribute the effects specifically to moringa. Both *C. longa* and *M. koenigii* independently demonstrated lipid-lowering and cardiometabolic benefits in clinical and preclinical settings [58,59]. Moreover, the addition of dietary counseling and exercise, known to improve lipid metabolism through enhanced lipoprotein lipase activity and increased HDL-C concentrations [60], further complicates interpretation. Therefore, it is plausible that the combination of regular exercise and botanical supplementation led to synergistic effects in lipid regulation in that trial, further complicating the attribution of observed benefits to moringa alone.

Moreover, even though the study by Afiaenyi et al. [30] provided a more focused evaluation of moringa direct effects on lipid outcomes, it was an unblinded, three-arm RCT in individuals with T2DM who were administered steamed moringa leaves at doses of 10, 20, and 30 g/day over two weeks. The authors reported dose-dependent reductions in TC and LDL-C, and increase in HDL-C and TAG levels, with the 20 g/day group exhibiting the most pronounced effects. While these results suggested a potential lipid-lowering effect of high-dose steamed moringa, the brief intervention duration and open-label design limit the strength of causal inferences. Furthermore, the use of steamed leaves raises important questions regarding compound stability and their bioaccessibility. Recent evidence from Wu et al. [61] demonstrates that steaming can significantly alter the structural and chemical properties of bioactive plant polysaccharides [61]. In their study on *Polygonatum cyrtonema* Hua, increased steaming duration reduced total carbohydrate content, increased uronic acids and (poly)phenols, and dramatically affected digestibility, fermentation, and microbiota composition. Longer steaming durations reduced the fermentability and short-chain fatty acid production of polysaccharides due to changes in molecular weight and monosaccharide composition, which influenced microbial utilization and bioactivity. These findings showed that steaming, although traditionally used to enhance palatability, may attenuate the metabolic effects of bioactive compounds by reducing their fermentability and altering gut microbiota interactions. In the context of Afiaenyi et al. [30] study, steaming may have modified the (poly)phenolic and fiber content of moringa, potentially reducing its prebiotic or lipid-modulating efficacy when compared to unprocessed or minimally processed preparations. This is particularly relevant given that moringa proposed lipid-lowering effects are linked to compounds like quercetin, chlorogenic acid, and saponins, many of which are heat-sensitive or bind to fiber matrices altered by thermal processing. Therefore, the observed “positive” effects might have been caused by other factors unrelated to moringa supplementation.

Furthermore, the study by Munir et al. [52] used 1 g/day of moringa leaves powder for 30 days in overweight individuals with hyperlipidemia and reported non-significant reductions across all lipid parameters despite an appropriate study population and elevated baseline lipid values. Similarly, Gómez-Martínez et al. [28] and Díaz-Prieto et al. [27], using a 2.4 g/day dose of moringa leaves powder in subjects with prediabetes, observed favorable within-group changes in glycemic markers but no significant changes in TAGs, TC, LDL-C, or HDL-C.

In addition to overall pooled results, subgroup analyses showed that doses <10 g/day, longer duration studies (≥12 weeks), and participants’ age (<50 years old) were associated with greater TAGs reductions when compared. This finding contrasts with the common assumption that higher doses yield stronger effects and may reflect issues related to bioavailability, tolerability, or adherence. Also, this trend suggests that chronic supplementation may be more effective than short-term interventions, aligning with known lipid metabolism dynamics.

HDL-C response was particularly heterogeneous. Subgroup analysis by age revealed a significant improvement in HDL-C among participants aged <50 years, while those ≥50 years experienced reductions, yielding a statistically significant difference. Similarly, participants receiving ≥10 g/day showed a significant decrease in HDL-C, whereas those consuming <10 g/day exhibited a positive but non-significant effect. These findings require confirmation through stratified analyses in future studies to elucidate potential age-related metabolic differences and dose-dependent absorption or efficacy thresholds.

Overall, while pooled estimates did not support a significant effect of moringa supplementation on lipid parameters, because of problems raised by the studies’ methodology, subgroup analyses suggested that dosage, duration, and participant age may influence lipid responses.

### 4.3. Moringa Supplementation and Glycaemic Outcomes

The meta-analysis also showed no statistically significant reductions in FBG or HbA1c following moringa supplementation. Nonetheless, individual trials varied notably with respect to intervention duration, population metabolic status, and moringa formulation.

For example, the double-blind trials by Gómez-Martínez et al. [28] and Díaz-Prieto et al. [27] in individuals with prediabetes reported reductions in both FBG and HbA1c after 12 weeks of supplementation with 2.4 g/day of moringa leaf powder. These findings, although not consistently replicated across studies, indicate that responses may differ according to dose and population characteristics. Similarly, Leone et al. [50] observed a decrease in HbA1c (−0.59%) in Sahrawi women with type 2 diabetes receiving 10 g/day of moringa leaves powder for three months. However, this study was unblinded and conducted under real-world conditions, which may introduce performance and detection bias. The absence of change in FBG despite HbA1c reduction may also reflect the inherent differences in these biomarkers, rather than a consistent treatment effect.

By contrast, Afiaenyi et al. [30] administered steamed moringa leaves (10–30 g/day) for two weeks in patients with type 2 diabetes and found no significant changes in FBG. The short duration and processing conditions may have influenced the bioactivity of the intervention. Collectively, these mixed findings emphasize the influence of methodological differences such as intervention length, formulation, and baseline metabolic status.

While several mechanistic studies proposed that moringa compounds could influence glucose metabolism through pathways including α-amylase and α-glucosidase inhibition [62], AMPK activation [63,64], or modulation of intestinal glucose transport [65], current clinical evidence does not allow firm conclusions regarding these mechanisms in humans. Heterogeneity in formulations, dosing, and concurrent lifestyle interventions further limits comparability among trials.

### 4.4. Moringa Supplementation and Blood Pressure

The pooled meta-analysis revealed that moringa supplementation had no significant effect on SBP, but was associated with a modest, statistically significant reduction in DBP. However, sensitivity analysis revealed that the significance of the DBP effect was not robust, as it was lost when individual studies were excluded. Subgroup analyses provided further insight into potential modifiers of the intervention’s effectiveness. Participants receiving <10 g/day of moringa experienced a significant reduction in DBP. These findings suggest that any antihypertensive effect of moringa may be limited, context-dependent, or attributable to specific subgroups.

Among the included trials, Afiaenyi et al. [30] reported a reduction in SBP (−9.16 mmHg) in the 20 g/day group after 14 days of steamed moringa leaf intake in patients with type 2 diabetes, though DBP changes were not significant. The short intervention period, unblinded design, and use of heat-processed leaves considerably limit the internal validity and external generalizability of these results. Moreover, thermal processing may alter the content of thermolabile (poly)phenols and glucosinolates [61], potentially affecting the biochemical profile of the intervention. Similarly, Taweerutchana et al. [31] administered 8 g/day of moringa capsules for four weeks in therapy-naïve individuals with type 2 diabetes and observed small, non-significant reductions in SBP and DBP (~−5 mmHg). While promising, the short duration and small sample size reduce the power to detect clinically meaningful differences. Díaz-Prieto et al. [27] used 2.4 g/day in prediabetic adults for 12 weeks and found no significant changes in blood pressure outcomes.

Variability among studies likely reflects differences in baseline blood pressure, sample size, intervention duration, and formulation type. Trials that included normotensive or low-risk participants may not have been able to detect measurable effects. Although mechanistic studies in animal models have suggested that moringa constituents can influence vascular tone through endothelium-dependent vasodilation, ACE inhibition [66], and antioxidant activity [67], these pathways have not been conclusively demonstrated in human populations.

Taken together, while moringa may exert modest DBP-lowering effects, the current evidence from human RCTs is limited by small sample sizes, short durations, inconsistent formulations, and methodological bias.

### 4.5. Strengths and Limitations

This meta-analysis presents several methodological and scientific strengths. It is the first systematic review and meta-analysis exclusively focused on RCTs assessing the effects of moringa supplementation on a broad range of cardiometabolic risk factors in adults. A comprehensive and systematic search strategy was implemented across three major databases, followed by rigorous screening and independent data extraction by multiple reviewers. The study was prospectively registered in PROSPERO and adhered to the PRISMA 2020 guidelines, ensuring transparency and reproducibility. Moreover, only RCTs with a minimum duration of 2 weeks and appropriate placebo were included, which increases internal validity. Another strength lies in the use of standardized effect size measures, subgroup and sensitivity analyses, and a detailed risk of bias assessment using the Cochrane RoB 2 tool. Also, we performed a GRADE assessment to systematically evaluate how methodological limitations and heterogeneity influenced the certainty of pooled estimates.

However, some limitations must be acknowledged. High between-study heterogeneity was observed across several outcomes, particularly anthropometric and lipid markers, which limits the confidence in pooled effect estimates. This reflects variability in study design, moringa formulation, dosage, intervention duration, and baseline participant characteristics. Although subgroup analyses provided a partial explanation for the observed heterogeneity, the small number of available RCTs did not permit meta-regression analyses to formally identify its sources. Moreover, only a limited number of subgroup analyses were conducted due to the small number of included studies, which prevented further exploration by study design (parallel vs. crossover), health status (healthy vs. cardiometabolic), intervention type (powder vs. fresh/steamed), or participant sex (male, female, mixed). In addition, several included trials were underpowered to detect small-to-moderate effects, further reducing the ability to draw firm conclusions. Intervention characteristics also varied widely, including the form of moringa used (leaves powder, steamed leaves, polyherbal combinations), dosage (900 mg/1 g to 30 g/day), and duration (2 weeks to 6 months), making direct comparisons challenging. Finally, several included studies were at high or unclear risk of bias, primarily due to insufficient reporting of randomization and allocation procedures, lack of blinding, and missing pre-registration. Additionally, the small number of eligible trials per outcome limited the ability to assess publication bias and raises the possibility that studies with null or negative results may be underrepresented in the literature.

### 4.6. Implications for Practice and Research

The findings of this meta-analysis suggest that moringa supplementation does not consistently improve major cardiometabolic parameters in adults, though subgroup trends point to potential benefits under specific conditions, namely, moderate dosing (<10 g/day), interventions lasting ≥12 weeks, and younger (<50 years) or metabolically at-risk populations. While reductions in DBP and improvements in HDL-C and TAGs were observed in some subgroups, these effects were either modest or not robust in sensitivity analysis. Given the generally favorable safety profile and accessibility of moringa, it may hold value as an adjunctive strategy, particularly in early-stage metabolic dysfunction or when integrated into broader lifestyle interventions. However, current evidence does not justify its use as a standalone therapeutic agent for managing cardiometabolic risk in clinical practice.

Future research should prioritize well-designed, adequately powered, placebo-controlled RCTs with standardized moringa formulations, detailed reporting of nutrient composition, and use of isocaloric controls. Trials should focus on populations with elevated cardiometabolic risk, employ clinically meaningful endpoints (e.g., HbA1c, lipid subfractions, vascular function), and incorporate mechanistic assessments, including glycemic variability, inflammatory biomarkers, and gut microbiota modulation. Dose–response studies are particularly needed to determine the optimal intake range and clarify whether higher doses are counterproductive due to caloric burden or bioavailability limitations. Ultimately, integrating moringa into evidence-based dietary frameworks, such as the Mediterranean or DASH diets, may offer a more practical and synergistic approach for improving cardiometabolic health.

## 5. Conclusions

Moringa is widely consumed in various forms across cultures due to its accessibility, nutritional density, and reputation as a functional food. Its leaves are rich in (poly)phenols, flavonoids, fiber, and micronutrients that have been mechanistically linked to glucose regulation, lipid metabolism, and vascular function. This systematic review and meta-analysis synthesized evidence from nine randomized controlled trials (12 study arms) including more than 600 participants. Overall, moringa supplementation did not produce statistically significant effects on most cardiometabolic parameters. A modest reduction in diastolic blood pressure was initially observed, though this effect was not robust in sensitivity analysis. Given the small number of available trials, high heterogeneity, and frequent methodological limitations, including inadequate blinding, short intervention durations, and lack of dietary control, the overall certainty of evidence was rated as very low across all outcomes according to the GRADE assessment. These limitations preclude firm conclusions regarding the efficacy of moringa supplementation for cardiometabolic risk reduction. As such, future high-quality trials should use standardized moringa preparations, matched isocaloric controls, and adequately powered designs targeting populations with elevated cardiometabolic risk. Until more definitive evidence emerges, moringa should not be recommended as a standalone strategy for cardiometabolic risk reduction but may be considered as part of a broader dietary and lifestyle framework.

## Figures and Tables

**Table 1:** Detailed PICOS framework in which the eligibility criteria for studies included in the systematic review and meta-analysis of *Moringa oleifera* supplementation and cardiometabolic outcomes in adults are provided.

Component	Criteria
Population (P)	Adults aged ≥18 years, irrespective of baseline health status. Studies on children, pregnant or lactating women were excluded.
Intervention (I)	*Moringa oleifera* supplementation in any form (e.g., leaves or fruit extracts, powders, capsules, tablets, or functional foods). Multi-component interventions were excluded unless the effect of moringa could be isolated.
Comparator (C)	(Isocaloric) placebo.
Outcomes (O)	At least one of the following cardiometabolic outcomes: − Anthropometric: body weight (BW), body mass index (BMI), waist circumference (WC). − Lipid profile: triacylglycerols (TAGs), total cholesterol (TC), LDL-C, HDL-C. − Glycemic: fasting blood glucose (FBG), insulinemia, HbA1c, HOMA-IR. − Blood pressure: systolic blood pressure (SBP), diastolic blood pressure (DBP).
Study Design (S)	Randomized controlled trials (RCTs), either parallel or crossover, with an intervention duration of ≥2 weeks. Non-randomized, observational, preclinical, or uncontrolled studies, and studies without sufficient outcome data were excluded.

**Table 2:** Characteristics of included randomized controlled trials (RCT) evaluating *Moringa oleifera* supplementation impact on cardiometabolic outcomes. Abbreviations: type 2 diabetes mellitus (T2DM), body weight (BW), body mass index (BMI), waist circumference (WC), triacylglycerols (TAGs), total cholesterol (TC), low-density lipoprotein cholesterol (LDL-C), high-density lipoprotein cholesterol (HDL-C), fasting blood glucose (FBG), glycosylated hemoglobin (HbA1c), homeostatic model assessment for insulin resistance (HOMA-IR), systolic blood pressure (SBP), diastolic blood pressure (DBP).

Study (Year), Country	Study Design	Participants Characteristics	Investigational Approach				Duration	Measured Outcomes
			Type	(*n*)	Intervention	Control		
Taweerutchana et al. (2017), Thailand [31]	Randomized, placebo-controlled, parallel trial	T2DM subjects Intervention: Age—52 ± 11 years BMI—28.1 ± 4.6 kg/m^2^ Control: Age—57 ± 7 years BMI—27.1 ± 3.2 kg/m^2^ Sex distribution: mixed (15 F/17 M)	Moringa leaves powder	Intervention: 16 Control: 16	4 g/d	Placebo (plain powder, magnesium stearate, and talcum)	4 wk	BW, FBG, HbA1c, SBP, DBP
Tshingani et al. (2017), Democratic Republic of Congo [49]	Randomized, single-blinded, placebo-controlled, parallel trial	HIV-infected subjects Intervention: Age—49.5 (21–64) years BMI—21.8 (4.2) kg/m^2^ Control: Age—47.0 (18–61) years BMI—21.4 (3.8) kg/m^2^ Sex distribution: mixed (61 F/19 M)	Moringa leaves powder	Intervention: 40 Control: 40	30 g/d (10.5 g proteins, 20.7 g lipids, 0.6 g carbohydrates, 232.5 kcal)	Standardized dietary counseling (healthy, balanced and energetic diet)	6 m	BMI
Dixit et al. (2018), India [29]	Randomized, double-blinded, placebo-controlled, parallel trial	Overweight subjects Intervention: Age—35.16 ± 9.29 years BMI—28.71 ± 0.89 kg/m^2^ Control: Age—37.26 ± 9.75 years BMI—28.41 ± 1.04 kg/m^2^ Sex distribution: mixed (82 F/58 M)	LI85008F formulae (6:3:1 hydro-ethanolic Moringa leaves extract, hydro-ethanolic *Murraya koenigii* leaves extract, and *C. longa* extract [≥95% curcuminoids])	Intervention: 70 Control: 70	900 mg/d (7.0% total curcuminoids, 0.1% mahanine, 0.2% quercetin 3-*O*-glycoside)	Placebo (99.56% corn starch, 0.44% syloid)	16 wk	BW, BMI, WC, TAGs, TC, LDL-C, HDL-C
Gambo et al. (2021), (2022), Nigeria [46,47,48]	Randomized, double-blinded, placebo-controlled, parallel trial	HIV-infected subjects Intervention: Age—~34.7 ± 9.0 years BMI—24.84 ± 4.76 kg/m^2^ Control: Age—~36.3 ± 9.3 years BMI—23.75 ± 3.82 kg/m^2^ Sex distribution: mixed (137 F/40 M)	Moringa leaves powder	Intervention: 89 Control: 88	15 g/d (4.2 g proteins, 0.585 g lipids, 3.3 g carbohydrates, 268.77 mg calcium, 731.89 mg potassium, 3.6 mg sodium, 0.432 mg zinc, 5.67 mg iron)	Placebo (cornstarch powder colored with chlorophyl)	6 m	BW, BMI
Gómez-Martínez et al. (2022), Díaz-Prieto et al. (2022), Spain [27,28]	Randomized, double-blinded, placebo-controlled, parallel trial	Prediabetic subjects Intervention: Age—56.2 ± 9.2 years BMI—29.4 ± 4.0 kg/m^2^ Control: Age—56.1 ± 10.8 years BMI—28.6 ± 3.8 kg/m^2^ Sex distribution: mixed (36 F/29 M)	Moringa leaves powder	Intervention: 31 Control: 34	2.4 g/d (0.67 g proteins, 0.11 g lipids, 1.18 g carbohydrates, 116.5 mg calcium, 66.3 mg potassium, 13 mg magnesium, 0.5 mg iron, 55.2 mg (poly)phenols)	Placebo (microcrystalline cellulose)	12 wk	BW, BMI, TAGs, TC, LDL-C, HDL-C, FBG, insulinemia, HbA1c, HOMA-IR, SBP, DBP
Sarfraz et al. (2023), Pakistan [51]	Randomized, placebo-controlled, parallel trial	Hyperlipidemic subjects Intervention: Age—25–55 years BMI—34.31 ± 6.04 kg/m^2^ Control: Age—25–55 years BMI—32.50 ± 8.86 kg/m^2^ Sex distribution: mixed (23 F/7 M)	Moringa leaves powder	Intervention: 15 Control: 15	2 g/d	Placebo (corn starch)	45 d	BMI, TAGs, TC, LDL-C, HDL-C
Afiaenyi et al. (2023), Nigeria [30]	Randomized, double-blinded, placebo-controlled, parallel trial	T2DM subjects Intervention: Age—63.80 ± 10.63 years BMI—23.41 ± 3.62 kg/m^2^ Control: Age—55.80 ± 9.26 years BMI—24.93 ± 4.24 kg/m^2^ Sex distribution: mixed (28 F/12 M)	Steamed Moringa leaves	Intervention: 30 Control: 10	20, 40, or 60 g/d	Isocaloric diet	2 wk	TAGs, TC, LDL-C, HDL-C, FBG, SBP, DBP
Munir et al. (2025), Pakistan [52]	Randomized, placebo-controlled, parallel trial	Overweight subjects Intervention: Age—44.15 ± 1.73 years BMI—31.79 ± 0.73 kg/m^2^ Control: Age—42.45 ± 1.74 years BMI—30.67 ± 0.05 kg/m^2^ Sex distribution: mixed (25 F/15 M)	Moringa leaves powder	Intervention: 20 Control: 20	1 g/d	Placebo (corn starch)	12 wk	BW, BMI, WC, TAGs, TC, LDL-C, HDL-C, FBG, SBP, DBP
Leone et al. (2025), Italy [50]	Randomized, unblinded, parallel trial	T2DM subjects Intervention: Age—56 ± 7 years BMI—30.4 ± 4.1 kg/m^2^ Control: Age—61 ± 7 years BMI—28.4 ± 4.9 kg/m^2^ Sex distribution: females (45)	Moringa leaves powder	Intervention: 30 Control: 15	10 g/d (2.70 g proteins, 0.58 g lipids, 1.48 g carbohydrates, 2.88 g fibers, 1.30 g ash, 0.23 g (poly)phenols)	Standard diet	3 m	BMI, WC, FBG, HbA1c

**Table 3:** GRADE summary of findings for the effects of *Moringa oleifera* supplementation on cardiometabolic parameters in adults. Abbreviations: body weight (BW), body mass index (BMI), waist circumference (WC), triacylglycerols (TAGs), total cholesterol (TC), low-density lipoprotein cholesterol (LDL-C), high-density lipoprotein cholesterol (HDL-C), fasting blood glucose (FBG), glycated hemoglobin (HbA1c), systolic blood pressure (SBP), diastolic blood pressure (DBP), randomized controlled trial (RCT), standardized mean difference (SMD), confidence interval (CI).

Outcome	No. of Studies	Risk of Bias	Inconsistency	Indirectness	Imprecision	Other Considerations	No. of Patients (I/C)	Effect (SMD, 95% CI)	Certainty
BW	5 RCTs	Serious ^a^	Very serious ^b^	Not serious ^d^	Serious ^e^	Few trials; publication bias cannot be excluded; no dietary control ^g^	220/219	−0.70 [−1.81, 0.40]	⬤◯◯◯ Very low
BMI	7 RCTs	Serious ^a^	Very serious ^b^	Not serious ^d^	Serious ^e^	Few trials; publication bias cannot be excluded; no dietary control ^g^	279/263	−0.69 [−1.59, 0.22]	⬤◯◯◯ Very low
WC	5 RCTs	Serious ^a^	Very serious ^b^	Not serious ^d^	Serious ^e^	Few trials; publication bias cannot be excluded; no dietary control ^g^	135/125	−0.18 [−1.53, 1.17]	⬤◯◯◯ Very low
TAGs	7 RCTs	Serious ^a^	Very serious ^b^	Not serious ^d^	Serious ^e^	Few trials; publication bias cannot be excluded; no dietary control ^g^	162/163	−0.14 [−0.71, 0.44]	⬤◯◯◯ Very low
TC	7 RCTs	Serious ^a^	Very serious ^b^	Not serious ^d^	Serious ^e^	Few trials; publication bias cannot be excluded; no dietary control ^g^	162/163	−0.20 [−0.76, 0.35]	⬤◯◯◯ Very low
LDL-C	7 RCTs	Serious ^a^	Very serious ^b^	Not serious ^d^	Serious ^e^	Few trials; publication bias cannot be excluded; no dietary control ^g^	162/163	−0.30 [−1.10, 0.51]	⬤◯◯◯ Very low
HDL-C	7 RCTs	Serious ^a^	Very serious ^b^	Not serious ^d^	Serious ^e^	Few trials; publication bias cannot be excluded; no dietary control ^g^	162/163	0.18 [−0.60, 0.96]	⬤◯◯◯ Very low
FBG	7 RCTs	Serious ^a^	Not serious ^c^	Not serious ^d^	Serious ^e^	Few trials; publication bias cannot be excluded; no dietary control ^g^	112/115	−0.12 [−0.38, 0.14]	⬤◯◯◯ Very low
HbA1c	3 RCTs	Serious ^a^	Not serious ^c^	Not serious ^d^	Serious ^e^	Few trials; publication bias cannot be excluded; no dietary control ^g^	77/65	−0.30 [−0.65, 0.05]	⬤◯◯◯ Very low
SBP	6 RCTs	Serious ^a^	Not serious ^c^	Not serious ^d^	Serious ^e^	Few trials; publication bias cannot be excluded; no dietary control ^g^	84/92	−0.14 [−0.47, 0.18]	⬤◯◯◯ Very low
DBP	6 RCTs	Serious ^a^	Not serious ^c^	Not serious ^d^	Serious ^f^	Few trials; publication bias cannot be excluded; no dietary control ^g^	84/92	−0.41 [−0.75, −0.07]	⬤◯◯◯ Very low

Footnotes: ^a^—Several studies rated as “some concerns” or “high risk”, see Figure 6 for more details; downgraded by one level. ^b^—Substantial heterogeneity (*I*^2^ ≥ 75%) and very heterogeneity (*I*^2^ ≥ 90%), with inconsistent direction of effects, see Figure 2 and Figure 3 for more details; downgraded by two levels. ^c^—Low heterogeneity (*I*^2^ < 20%), consistent direction of effect, see Figure 4 and Figure 5 for more details; not downgraded. ^d^—Populations and interventions align with review question, see Table 1 and Table 2 for more details. Not downgraded. ^e^—Wide CIs spanning potential benefit and null effect. Downgraded by one level. ^f^—Result fragile; significance lost in leave-one-out analysis. Downgraded by one level. ^g^—All outcomes were evaluated by a small number of trials (<10), preventing reliable assessment of publication bias; additionally, the absence of dietary monitoring or control in most studies may have influenced the observed effects. Downgraded by one level.

## Data Availability

The authors confirm that the data supporting the findings of this study are available under request.

## Supplementary Materials

The following supporting information can be downloaded at: https://www.mdpi.com/article/10.3390/nu17223501/s1, Supplementary Table S1. Search strategies including the key terms and the queries for each database; Supplementary Table S2. Results of subgroup analysis of included studies in the meta-analysis; Supplementary Table S3. Detailed justifications for the risk of bias judgments across all domains for each included study.
