# Supplementary material for: Effects of Moringa oleifera Lam. Supplementation on Cardiometabolic Outcomes: A Meta-Analysis of Randomized Controlled Trials with GRADE Assessment
Source: Nutrients. 2025 Nov 7;17(22):3501. doi: 10.3390/nu17223501 (PMC12655524; doi:10.3390/nu17223501)
Supplement: Supplementary file 1 [file nutrients-17-03501-s001.zip › nutrients-3967942-supplementary.pdf]

Supplementary Materials

Table S1. Search strategies including the key terms and the queries for each database.

Database	Key terms and the queries
PubMed	("Moringa oleifera"[Mesh] OR "Moringa oleifera" OR "Moringa") AND ("randomized controlled trial" OR "randomised controlled trial" OR randomized OR randomised OR RCT OR trial) AND "humans"[MeSH Terms]
WebOfScience	TS=("Moringa oleifera" OR Moringa) AND TS=("randomized controlled trial" OR "randomised controlled trial" OR randomized OR randomised OR RCT OR trial) AND DT=(Article) NOT DT=(Review)
Scopus	TITLE-ABS-KEY ("Moringa oleifera" OR moringa) AND TITLE-ABS-KEY ("randomized controlled trial" OR "randomised controlled trial" OR randomized OR randomised OR rct OR trial) AND TITLE-ABS-KEY ("body weight" OR "BMI" OR "waist circumference" OR "fasting glucose" OR insulin OR "HbA1c" OR "HOMA-IR" OR cholesterol OR triglycerides OR "lipid profile" OR "blood pressure" OR "systolic blood pressure" OR "diastolic blood pressure")

Table S2. Results of subgroup analysis of included studies in the meta-analysis. Abbreviations: body weight (BW), body mass index (BMI), waist circumference (WC), triacylglycerols (TAG), total cholesterol (TC), low-density lipoprotein cholesterol (LDL-C), high-density lipoprotein cholesterol (HDL-C), fasting blood glucose (FBG), systolic (SBP) and diastolic blood pressure (DBP).

Sub-grouped by	No. of trials	Effect size[1]	95% CI, P value	I^2 (%)	P for heterogeneity	P for between subgroup heterogeneity
**BW (All trials)**	**5**	**-0.70**	**[-1.81, 0.40], 0.21**	**96**	**<0.00001**	
Age						0.22
<50	3	-1.17	[-2.97, 0.62], 0.20	98	<0.00001	
≥50	2	-0.01	[-0.42, 0.40], 0.96	0	0.75	
**BMI (All trials)**	**7**	**-0.69**	**[-1.59, 0.22], 0.14**	**95**	**<0.00001**	
Dosage						0.07
<10 g/day	4	-1.31	[-2.80, 0.17], 0.08	96	<0.00001	
≥10 g/day	3	0.07	[-0.17, 0.32], 0.56	4	0.35	
Age						0.18
<50	5	-0.95	[-2.21, 0.31], 0.14	97	<0.00001	
≥50	2	-0.05	[-0.44, 0.35], 0.82	0	0.85	
BMI						0.08
<25	2	0.13	[-0.27, 0.54], 0.52	51	0.15	
≥25	5	-1.05	[-2.31, 0.22], 0.10	95	<0.00001	
**WC (All trials)**	**4**	**-0.18**	**[-1.53, 1.17], 0.79**	**95**	**<0.00001**	
Age						0.77
<50	2	-0.46	[-3.77, 2.85], 0.79	99	<0.00001	
≥50	2	0.03	[-0.40, 0.46], 0.90	0	0.71	
**TAG (All trials)**	5	**-0.14**	**[-0.71, 0.44], 0.64**	**82**	**<0.00001**	
Dosage						0.002
<10 g/day	4	-0.60	[-1.16, -0.04], 0.04	77	0.004	
≥10 g/day	1	0.07	[-0.17, 0.32], 0.56	4	0.35	
Duration						0.02
<12 wk	2	0.33	[-0.19, 0.85], 0.21	32	0.003	
≥12 wk	3	-0.69	[-1.37, -0.01], 0.05	83	<0.00001	
Age						0.002
<50	3	-0.81	[-1.32, -0.29], 0.002	60	0.08	
≥50	2	0.31	[-0.15, 0.78], 0.19	33	0.21	
**TC (All trials)**	5	-0.20	[-0.76, 0.35], 0.47	81	<0.00001	
Dosage						0.03
<10 g/day	4	-0.55	[-1.19, 0.09], 0.09	83	0.0006	
≥10 g/day	1	0.36	[-0.15, 0.87], 0.17	0	0.77	
Duration						0.10
<12 wk	2	0.14	[-0.28, 0.56], 0.52	0	0.44	

≥12 wk	3	-0.62	[-1.41, 0.17], 0.13	87	0.0004	
Age						0.07
<50	3	-0.68	[-1.44, 0.07], 0.08	81	0.005	
≥50	2	0.08	[-0.28, 0.43], 0.66	0	43	
**LDL-C (All trials)**	5	-0.30	[-1.10, 0.51], 0.47	91	<0.00001	
Dosage						0.07
<10 g/day	4	-0.73	[-1.77, 0.31], 0.17	93	<0.00001	
≥10 g/day	1	0.34	[-0.21, 0.88], 0.22	10	0.33	
Duration						0.09
<12 wk	2	0.20	[-0.22, 0.62], 0.34	0	0.40	
≥12 wk	3	-0.95	[-2.20, 0.31], 0.14	94	<0.00001	
Age						0.06
<50	3	-0.98	[-2.14, 0.17], 0.09	91	<0.00001	
≥50	2	0.16	[-0.20, 0.52], 0.39	4	0.37	
**HDL-C (All trials)**	5	0.18	[-0.60, 0.96], 0.65	90	<0.00001	
Dosage						0.02
<10 g/day	4	0.72	[-0.23, 1.67], 0.14	92	<0.00001	
≥10 g/day	1	-0.62	[-1.14, -0.10], 0.02	0	95	
Duration						0.11
<12 wk	2	-0.30	[-0.90, 0.30], 0.32	49	0.12	
≥12 wk	3	0.80	[-0.43, 2.02], 0.20	94	<0.00001	
Age						<0.00001
<50	3	1.10	[0.41, 1.78], 0.002	75	0.02	
≥50	2	-0.44	[-0.80, -0.08], 0.02	0	0.80	
**FBG (All trials)**	5	-0.12	[-0.38, 0.14], 0.38	0	0.69	
Duration						0.08
<12 wk	3	0.10	[-0.25, 0.46], 0.58	0	0.98	
≥12 wk	2	-0.38	[-0.76, 0.01], 0.06	0	0.56	
Age						0.10
<50	3	-0.30	[-0.64, 0.04], 0.08	0	0.62	
≥50	2	0.15	[-0.26, 0.56], 0.47	0	0.98	
BMI						0.79
<25	2	-0.14	[-0.52, 0.24], 0.46	8	0.35	
≥25	3	-0.07	[-0.46, 0.32], 0.73	0	0.76	
**SBP (All trials)**	4	-0.14	[-0.47, 0.18], 0.39	14	0.32	
Dosage						0.05
<10 g/day	3	-0.37	[-0.74, 0.00], 0.05	0	0.41	
≥10 g/day	1	0.26	[-0.25, 0.77], 0.32	0	0.89	
Duration						0.32
<12 wk	2	0.01	[-0.40, 0.43], 0.95	0	0.43	
≥12 wk	2	-0.35	[-0.92, 0.23], 0.24	42	0.19	
**DBP (All trials)**	4	-0.41	[-0.75, -0.07], 0.02	19	0.29	
Dosage						0.02
<10 g/day	3	-0.70	[-1.08, -0.32], 0.0003	0	0.70	
≥10 g/day	1	0.05	[-0.45, 0.56], 0.84	0	0.98	
Duration						0.20
<12 wk	2	-0.22	[-0.70, 0.26], 0.37	25	0.26	
≥12 wk	2	-0.65	[-1.09, -0.20], 0.004	0	0.48	

Table S3. Detailed justifications for the risk of bias judgments across all domains for each included study.

Study (First Author, Year)	Domain 1: Randomization	Justification	Domain 2: Deviations	Justification	Domain 3: Missing Data	Justification	Domain 4: Measurement	Justification	Domain 5: Reporting	Justification	Overall RoB
**Ghiridhari, 2011**	Some concerns	No info on allocation concealment; moderate baseline imbalance	Some concerns	No blinding reported, placebo not clearly matched	Low risk	All participants completed	Low risk	Objective outcomes used	Some concerns	No protocol or analysis plan	Some concerns
**Taïrou, 2022**	Some concerns	Randomization method unclear; baseline albumin/CRP imbalanced	Some concerns	Single-blind; participant not blinded	High risk	Only 60/80 randomized analyzed; exclusion unexplained	Low risk	Lab outcomes used, blinded assessors	Some concerns	Retrospective registration	High risk
**Unnikrishnan, 2021**	Low risk	Computer-generated block randomization, proper concealment	Low risk	Double-blind, well-controlled setting	Low risk	<10% attrition, balanced	Low risk	Objective outcomes and assessor blinding	Low risk	Trial was pre-registered and followed	Low risk
**Ibe, 2023**	Low risk	Urn algorithm, concealment maintained by statistician	Low risk	Double-blind; good compliance monitoring	Low risk	Balanced, <15% attrition, unrelated to outcomes	Low risk	Lab measurements, blinded assessors	Low risk	Registered, outcomes consistent with plan	Low risk
**Gómez-Martínez & Díaz-Prieto, 2021–2022**	Low risk	Block randomization described, good baseline comparability	Low risk	Double-blind; adherence confirmed	Low risk	~11% dropout, balanced and justified	Low risk	Central lab, objective outcomes	Low risk	All outcomes per registry reported	Low risk
**Ifeoma, 2021**	Some concerns	Envelope method used; no concealment; baseline imbalance	Low risk	Controlled meals and supplement administration	Low risk	ITT with last observation carried forward	Low risk	Outcomes objective; blinding likely	Low risk	Retrospective registration, but matched outcomes	Some concerns
**Sarfraz, 2023**	High risk	"Random sampling" with no allocation method; baseline	High risk	No blinding of participants or assessors	Low risk	No dropout or missing data	Some concerns	Objective outcomes, but assessors not blinded	Some concerns	No trial registration	High risk

		imbalance									
**Leone, 2025**	Some concerns	Sequence used, but concealment not reported; retrospective registration	High risk	Participants were unblinded, risk of behavioral influence	Low risk	Minor attrition, balanced between groups	Some concerns	Objective lab data; blinding not reported	Some concerns	Retrospectively registered	High risk
**Munir, 2025**	Some concerns	"Random assignment" without method or concealment details	High risk	No mention of blinding; dietary & physical activity could be biased	Low risk	All participants completed the study	Some concerns	Objective outcomes, no info on assessor blinding	Some concerns	Unregistered; selective reporting cannot be ruled out	High risk
